# Amphiphilic DNA Organic Hybrids: Functional Materials in Nanoscience and Potential Application in Biomedicine

**DOI:** 10.3390/ijms19082283

**Published:** 2018-08-03

**Authors:** Zhiyong Zhao, Ting Du, Feng Liang, Simin Liu

**Affiliations:** The State Key Laboratory of Refractories and Metallurgy, School of Chemistry and Chemical Engineering, Wuhan University of Science and Technology, Wuhan 430081, China; zhaozhiyong@wust.edu.cn (Z.Z.); tingdu201222@hotmail.com (T.D.)

**Keywords:** small molecule modified DNA, DNA block copolymers, DNA-dendron hybrids, self-assembly, nanostructure, nanoscience, biomedicine

## Abstract

Due to the addressability and programmability, DNA has been applied not merely in constructing static elegant nanostructures such as two dimensional and three dimensional DNA nanostructures but also in designing dynamic nanodevices. Moreover, DNA could combine with hydrophobic organic molecules to be a new amphiphilic building block and then self-assemble into nanomaterials. Of particular note, a recent state-of-the-art research has turned our attention to the amphiphilic DNA organic hybrids including small molecule modified DNA (lipid-DNA, fluorescent molecule-DNA, etc.), DNA block copolymers, and DNA-dendron hybrids. This review focuses mainly on the development of their self-assembly behavior and their potential application in nanomaterial and biomedicine. The potential challenges regarding of the amphiphilic DNA organic hybrids are also briefly discussed, aiming to advance their practical applications in nanoscience and biomedicine.

## 1. Introduction

In nature, every life form possesses its own multi-level and ordered structure. The highly functional nanostructures have attracted much research interest from chemists. These supramolecular structures are built from the self-assembly of nucleic acids, proteins, and other (macro)molecular building blocks through molecular recognition such as hydrogen bonding, metal coordination, hydrophobic forces, π-π interactions, and other noncovalent interactions. Inspired by the self-assembly behavior in biological system, lots of scientists devote themselves to exploiting biomimetic materials. An important and present mature research method to construct the diverse nanoarchitectures with dimensions in the range of 5 to 100 nm is bottom-up strategy.

In addition to being a genetic material, oligonucleotide is also a useful bottom-up building material to construct one dimensional (1D), two dimensional (2D), and three dimensional (3D) DNA nanostructures [1,2,3,4,5,6] and dynamic nanodevices [7,8,9,10], due to its unique properties, e.g., specific Watson-Crick base pairing recognition, designable sequence, and reliable synthesis. Moreover, oligonucleotide could be functionalized with different agents and then utilized in nanotechnology and biomedicine. Oligonucleotide also could be functionalized with inorganic materials such as gold nanoparticles [11,12], organic molecules, and other biologic macromolecules like proteins [13]. With the development of the chemical modification technology of DNA, the amphiphilic DNA organic hybrids can be synthesized from hydrophobic molecules and DNA through solid-phase synthesis [14]. These hydrophobic molecules include lipid, porphyrin and pyrene, synthetic polymers, and dendrons. The amphiphilic organic hybrids can be regarded as new building blocks, and then spontaneously assembled into various nanostructures in aqueous solution, e.g., spherical micelles, nanofibers, bilayers, and vesicles. Because these component molecules can possess intrinsic functionality, such as redox, photophysical, and biological properties, they afford the rational design of complex function into the final assemblies.

**Scheme 1:**
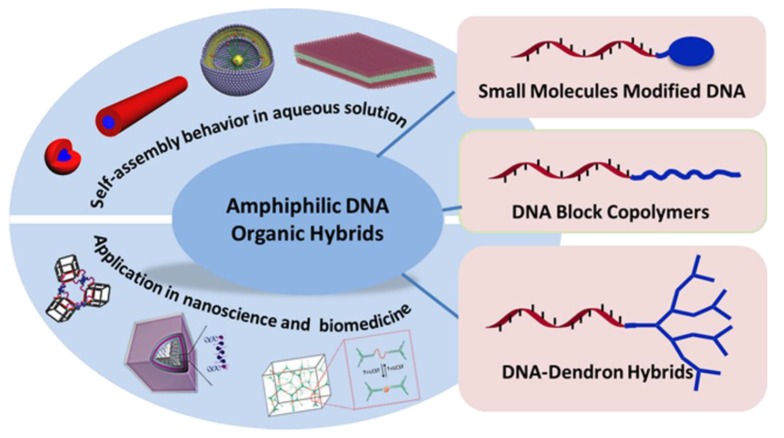
Illustration of self-assembly and application of the amphiphilic DNA organic hybrids, which includes small molecules modified DNA, DNA block copolymer, and DNA-dendron hybrids. The blue color in the amphiphilic DNA organic hybrids indicates the organic molecule and the deep red color chain indicate the DNA strand.

Until now, there have been many topic reviews reported about the terminal functionalization of DNA [15], which mainly introduced the solid-phase synthesis and solution coupling reaction methods for covalent terminal functionalization of DNA, and about the progress on DNA block copolymers [16,17,18,19,20], which stated the synthesis, self-assembly, and application of DNA block copolymers. However, small molecule modified DNA also can be regarded as an amphiphilic molecule and further undergo supramolecular assembly. Moreover, the development of the DNA block copolymers and DNA-dendron hybrids has expanded in biomedicine and nanoscience. Herein, this review will present a systematic introduction of the recent progress on the three kinds of amphiphilic DNA organic hybrids including small molecule modified DNA, DNA block copolymers, and DNA-dendron hybrids, as shown in Scheme 1. Their self-assembly and application in nanoscience and biomedicine will be demonstrated respectively. Finally, the conclusion and prospective will be discussed.

## 2. Small Molecules Modified DNA

DNA has shown its unique properties. When it combined with some specific molecules, the new hybrids reveal more interesting performances. As the development of the chemical modification of DNA, some small hydrophobic molecules could be decorated at an arbitrary position of the oligonucleotide, such as at the middle or at the end, to achieve their function. The different synthetic methods have been reported in several topical reviews [15,16]. Small molecules modified DNA hybrids have gained wide attention as tools for nucleic acid research, light harvesting, energy and electron transfer, sensing, diagnostics, and nanotechnology. The various approaches show that the diversity of available functionalities have been reviewed independently [21], in particular using organic chromophores [22], which shall not be covered here in detail. We will focus on lipid-DNA conjugates, pyrene modified DNA, and perylenediimide modified DNA with these building blocks to construct functional nano-assemblies, and further, to realize their different functionality.

### 2.1. Self-Assembly of Small Hydrophobic Molecule Modified DNA

#### 2.1.1. Lipid-DNA Conjugates

**Figure 1:**
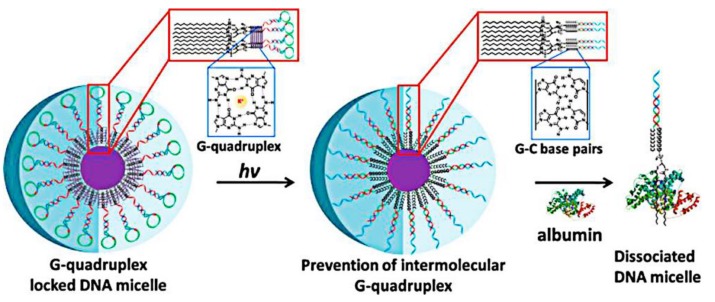
Schematic illustration of stability-tunable DNA–lipid micelles. DNA strand contains a G-rich sequence, which could form intermolecular G-quadruplex and dissociate at UV light. G-quadruplex stabilizes DNA micelles against disruption by serum albumin. While upon exposure to UV light, and adding complementary DNA to prevent the formation of G-quadruplex, the DNA–lipid micelles were dissociated in serum albumin. Purple core inside of the micelle stands for hydrophobic lipid core. Reproduced with permission from authors of a previous paper [28]. Copyright 2017 American Chemical Society.

Amphiphilic DNA-lipid conjugates have been synthesized for the antisense and membrane anchor applications [23]. DNA-lipid conjugates, as emerging building blocks, recently were used to construct supramolecular assemblies [24]. In 2006, Dentinger and colleagues reported that a tetradecyl hydrophobic tail was covalently attached to the synthetic oligomers to obtain the amphiphilic DNA hybrids. They could spontaneously assemble into vesicular aggregates to stabilize the hydrophobic molecules and release these hydrophobic molecules into aqueous solution via DNA hybridization [25]. In 2010, Tan group reported highly stable, monodispersed micelles from DNA–diacyllipid conjugates [23]. Their preliminary test showed that the DNA–lipid micelles possessed size-dependent cell permeability and could be internalized by endocytosis. In the same year, Gianneschi group introduced the responsive liposomes assembled from DNA–double chain lipid conjugates [26]. The liposomes with a diameter of 500 nm were capable of reversibly switching to 20–25 nm spherical micelles upon base pairing to a complementary DNA sequence. In 2016, Yang group reported the assembly behavior of DNA–lipid conjugates at the liquid crystal (LC)-aqueous interface. It proved that DNA–lipid conjugates could coassemble with l-dilauroylphosphatidylcholine at LC-aqueous interface, which resulted in the net-like structures in micron scale. By DNA hybridization, DNA–lipid conjugates desorbed from LC-aqueous interface into aqueous, resulting in the disappearance of net-like structures [27]. In 2017, Tan group developed the stability-tunable DNA micelles from DNA-lipid conjugates [28]. As shown in Figure 1, the DNA sequence contains the photocontrollable dissociation of intermolecular G-quadruplexes, which confers the DNA micelles with robust structural stability against disruption by serum albumin. However, the instability would occur once exposed to light as G-quadruplex formation is blocked by strand hybridization in the presence of the serum albumin and subsequent cellular uptake. With a similar idea, Roelfes and coworkers recently achieved the fabrication DNA–lipid micelles with G-quadruplex structures. The micelles were disaggregated by hybridization with a complementary DNA sequence and lead to cargo release [29].

**Figure 2:**
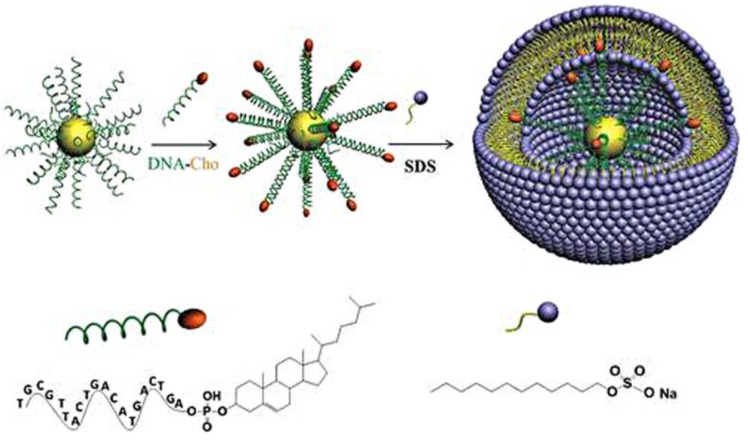
Schematic of formation of hetero-vesicles through the frame guided assembly process. Firstly, the gold nanoparticles were modified with 20-mer and 6-mer ssDNA. Then, DNA-cholesterol conjugates as leading hydrophobic groups were anchored to gold nanoparticles by base pairing interaction. Finally, the cholesterol molecules of the frame guided the assembly of amphiphilic SDS molecules. Reproduced with permission from the authors of a previous paper [32]. Copyright 2015 John Wiley and Sons Publisher.

Besides the self-assembly of pristine DNA–lipid conjugates, it can be mixed or covalent with other agents such as DNA nanostructures [30] or proteins [31] to assemble into hetero-nanostructures. In 2015, Liu and colleagues utilized the amphiphilic DNA-cholesterol conjugates as the leading hydrophobic groups to induce the guided assembly of amphiphiles sodium dodecyl sulfate (SDS) on the gold nanoparticles, with results of the formation of hetero-vesicles with different sizes and shapes [32], as shown in Figure 2. Subsequently, Beales and Vanderlick reported a novel reversible assembly of the stacked membrane nanodiscs from lipids, nucleic acids, and proteins [31]. Lipids firstly self-assembled into bilayer discs, stabilized by scaffold proteins. Lipid–DNA conjugates worked as molecular glues as the lipid segment was inserted into the bilayer nanodiscs and the DNA sequences was out of the membrane surface to hybridize with their complementary strands. After adding complementary DNA sequences, the bilayer nanodiscs were assembled into columnar membranes tacks with a periodic structure. This architecture is favorable due to its relative orientations of DNA and the shape anisotropy of bilayer discs. The BioNanoStacks are formed from the nanoscale two-dimensional membrane discs. The diameters of such materials with reduced dimensionality are dominated by the scaffold protein. Moreover, those materials assembled into a quasi-one dimensional strings by the DNA.

#### 2.1.2. Pyrene-Modified DNA

Pyrene has unique fluorescent properties of long fluorescence life time, high quantum yield, a fair range of sensitivity of fluorescence to the microenvironment, and excellent ability to form both excimers and exciplexes. Moreover, it can be used as a π-π stacking (including anchoring) moiety and be intercalated into nucleic acid duplex. Based on these properties, pyrene has been used as the agents for the binding of double-stranded DNA, aptamer-based biosensor, and the construction of sensitive fluorescent probes for the discrimination of single nucleotide polymorphisms (SNPs) and the sequence-specific detection of nucleic acids [33,34]. Here pyrene modified DNA hybrids as building blocks are capable to form supramolecular complexes. In 2015, Häner group developed that the chimeric oligomers composed of a heptapyrenotide part and an appended DNA strand were assembled into helical nanoribbons, driven by aromatics tacking interactions among pyrene units [35]. The nanoribbons could be changed to extended networks via DNA hybridization [35,36]. Using a similar protocol, they further proved that the helical supramolecular assemblies from the chimeric oligomers were able to load cargo or gold nanoparticles [37], which showed their potential application in drug delivery. Moreover, Häner group demonstrated the co-existence of two independent excitonic states in aromatic stacks composed of two types of chromophores. In a DNA scaffold, an alternating fashion were arranged by pyrene and perylenediimide (PDI) molecules. The observation of electronic coupling between chromophores of the same type in alternating arrangements indicated that the pyrene H-aggregates and PDI co-exist in p-stacked hetero-aggregates [38].

#### 2.1.3. Perylenediimide-Modified DNA

Another class of attractive chromophores is perylenediimide (PDI). It has been widely used as dye sensitizers in solar cells due to their outstanding thermal, chemical, and photochemical stability and high fluorescence intensity. Moreover, water soluble PDI molecules with biocompatibility and photostability have been successfully applied in the biological field [39]. Recently, PDI molecules were also attached to the DNA strand, at different locations [40,41,42,43,44]. Lewis and coworkers reported that PDI-linked bis(oligonucleotide) conjugates could form a hairpin dimer structure, driven by the hydrophobic interaction among PDI molecules [45]. Subsequently, Lewis and Rybtchinski further investigated the DNA dumbbell conjugates possessing hydrophobic PDI linkers. Because of the hydrophobic association of PDI molecules, the PDI-linker DNA dumbbell conjugates were assembled into linear end-to-end supramolecular polymers in diluted aqueous solution, and finally assembled into higher-order hexagonal-type aggregates after slow evaporation [46]. In 2017, Lewis introduced in detail the self-assembly of PDI-single strand DNA conjugates with different DNA lengths and different DNA sequences. These PDI–DNA conjugates in aqueous solution were assembled into a diverse structural space that includes fibers and more complex aggregates, depending on the hydrophobic π-π-stacking interaction of PDI molecules and base-pairing recognition of oligonucleotide [47]. These results provided a possible research approach to control over self-assembly and realizing novel structural motifs.

#### 2.1.4. Other Small Hydrophobic Molecule-DNA Conjugates

Porphyrin possesses the unique electronic properties which can be tailored through chemical modifications of the aromatic core or through insertion of various metals into the central cavity. So porphyrin modified DNA by covalent conjugation is regarded as a new building block in supramolecular chemistry. Strategies for the synthesis, characterization, and the developments for the construction of functional assembles were summarized in a topic review [48]. Besides, Das group reported the facile formation of nanocages from DNA–protoporphyrinIX (PpIX) hybrids [49]. The DNA–PpIX hybrids were synthesized through covalent linkage of the carboxyl groups of protoporphyrin IX with the amine groups of modified DNA strands. One protoporphyrin IX molecule was attached two strands of DNA, another protoporphyrin IX molecule was attached two strands of complementary DNA. During hybridization at 250 mM NaCl solution, the higher ordered 2D nanocages were generated through self-assembly. The nanostructures could be potentially used in coherent ROS generation, biochip fabrication and also to hold drug molecules, proteins or other nanoparticles.

**Figure 3:**
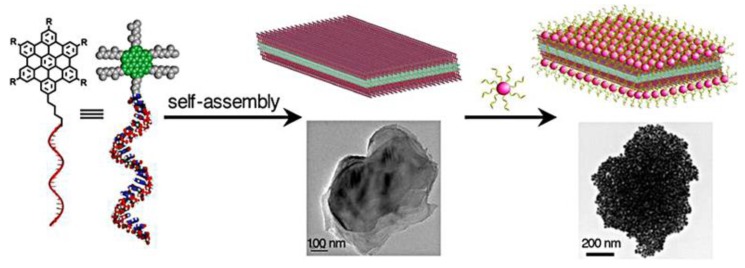
Illustration of the self-assembly of the amphiphilic DNA covalent with hexa-peri-benzocoronene tethered with alkyl chains. The amphiphiles assembled into 2D DNA nanosheets decorated with DNA sequences, which could further hybridize with complementary DNA modified gold nanoparticles. The green color indicates the hexa-peri-benzocoronene and the grey chains indicate the alkyl chains. Reproduced with permission from the authors of a previous paper [50]. Copyright 2017 American Chemical Society.

What morphology will form when the DNA-hydrophobic molecule hybrid composes of the larger aromatic compound and oligonucleotide? Varghese and colleagues stated that DNA-amphiphiles composed of hexa-peri-benzocoronene tethered with the alkyl chains and DNA were self-assembled into DNA-decorated, crystalline, 2D sheets [50], as shown in Figure 3. This work demonstrated that, irrespective of the rigidity and the length of hydrophilic DNA sequence, the assembly into sheets is essentially dictated by the strong self-assembling property of the hydrophobic segment of the amphiphiles. Hence, by choosing strongly π-stacking moiety as the hydrophobic fragment, this approach can be used as a general strategy to fabricate DNA-based micrometer-sized sheets.

More interestingly, Varghese group recently exploited a noncovalent method based on the host–guest interaction between β-CD and adamantane to construct DNA amphiphiles, and reported their self-assembly into DNA decorated vesicles [51]. The results suggested that the noncovalent approach could be one of the most simple, universal, and efficient methods for the generation of DNA supra amphiphiles, and provided an excellent strategy for the creation of smart DNA nanostructures.

### 2.2. Application of Small Molecule Modified DNA

These small hydrophobic molecule-DNA conjugates have been widely applied in different fields like quantification and purification of nucleic acids, sensors and assemblies for cargo release. In many applications, DNA–lipid conjugates are used to tag vesicles and/or membranes, which were summarized in some previous review articles [24,52]. Here we will focus on the recent development of DNA–lipid conjugates and their assemblies.

The lipid–DNA conjugates can be utilized to fuse liposomes, which were reported in two recent papers [53,54]. Kors and Herrmann reported the liposomes aggregation and liposomes fusion through the DNA hybridization. The lipids of the DNA–lipids acted as the anchors into the liposomes, and the DNA sequences were anchored at the surface of liposomes to further hybridize with complement, resulting in membrane fusion. It is noteworthy that the number and position of lipids of the DNA–lipid conjugates could be precisely controlled by automated solid phase synthesis, and in the most efficient system, it achieved about 30% full fusion [53]. Using the similar strategy, Vogel group have systematically investigated the ability of lipid–DNA conjugates to mediate and control the programmable fusion of liposomes by DNA hybridization. They studied the effect of different membrane anchor moieties, and temperature dependency of the fusion and content mixing process [54]. The DNA-hybridization-based liposome fusion and content mixing has useful application in investigating encapsulated processes.

The DNA–lipid assemblies have potential application in drug delivery. In 2010, Herrmann and Cornelissen utilized DNA–lipids micelles and DNA block copolymer micelles as the templates to assemble and load Cowpea Chlorotic Mottle Virus capsids [55]. The artificial virus-like particles were capable of copackaging with various small compounds by either hybridizing them onto the micelles or incorporating them into the hydrophobic core. The loading approach based on the virus-like particles is probably to be utilized as a high-impact drug delivery system. Similar to the reported results [28,29], the DNA–lipid assemblies with G-quadruplex structures were stable enough in the presence of the serum albumin and the subsequent cellular uptake was achieved through DNA hybridization, which suggested these biocompatible DNA micelles as nanocarriers for cargo release in vivo applications.

The other small hydrophobic molecules modified DNA assemblies could also be applied as nano-vehicles in biomedicine, mainly attributing to their properties as follows: the hydrophobic domain of the micelles or vesicles could envelope water insoluble molecules like drugs and hydrophobic fluorescent molecules applied in bioimaging and tracer techniques, and the DNA strands at the peripheral possess the ability to loading various functional structures by DNA hybridization.

## 3. Amphiphilic DNA Block Copolymers

Due to the inhere sequence programmability and base-pairing fidelity, DNA could be regarded as a polymer to be conjugated with synthesized hydrophobic polymer to obtain a new building block–an amphiphilic DNA block copolymer. Combined with the diversity and multi-responsiveness of synthetic polymers, the amphiphilic DNA block copolymers will achieve their specific functions. The synthesis method and some assembly behavior were introduced in detail in some excellent reviews [15,16,17,18,19,20], so here we mainly focus on the recent development.

### 3.1. Self-Assembly of Amphiphilic DNA Block Copolymers

Since the Park group [56] and Mirkin group [14] have reported the synthetic method and assembly behavior of the amphiphilic DNA block copolymers successively, most researchers have devoted to investigate the assembly of the different kinds of polymers covalent with single strand DNA [57,58,59,60,61]. Among them, Herrmann and colleagues have carried out lots of researches on the assembly process and the application of DNA block poly(propyleneoxide) (DNA-*b*-PPO) [57,62,63].

**Figure 4:**
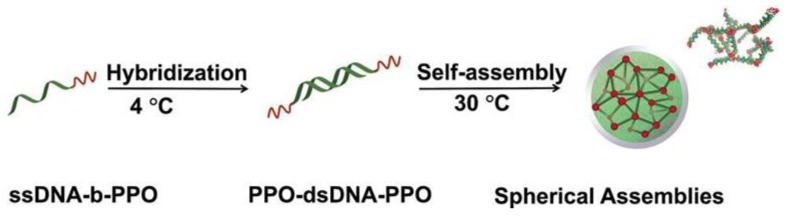
Illustration of the preparation and self-assembly of supramolecular triblock copolymer PPO-dsDNA-PPO. The coil-rod-coil supramolecular triblock copolymer PPO-dsDNA-PPO was firstly generated by DNA base pairing interaction at low temperature. Then by elevating the temperature, the triblock copolymers spontaneously assembled into large spherical micelles. The green chain represents DNA strand and the red chain represents PPO polymer. Reproduced with permission from the authors of a previous paper [64]. Copyright 2015 American Chemical Society.

More recently, the smart DNA block copolymer assemblies has been greatly investigated by several groups. The size and morphology of assemblies can be controlled by DNA hybridization or by external stimuli such as temperature, pH value, and ionic strength. In 2012, Liu group developed a pH-responsive morphology-controlling of DNA-*b*-PPO assemblies [59]. The amphiphilic DNA-*b*-PPO copolymers were assembled into 20 nm spherical micelles at pH 8.0 and assembled into long nanofibers at pH 5.0. The formation of spherical micelles and nanofibers was attributed to the folding and disassociating of a bimolecular “*i*-motif” structure at pH 5.0 and 8.0, respectively. Moreover, these shapes can be trans formed reversibly in situ by regulating temperature and pH value. Liu group further utilized DNA-*b*-PPO to prepare supramolecular triblock copolymers PPO-dsDNA-PPO by DNA hybridization [64]. As shown in Figure 4, the PPO-dsDNA-PPO triblock copolymers were assembled into spherical micelles with a diameter of 200 nm through elevating temperature. This system is different from the usual DNA diblock copolymers, therefore the mechanism of the self-assembly was further explained by molecular dynamic simulations.

**Figure 5:**
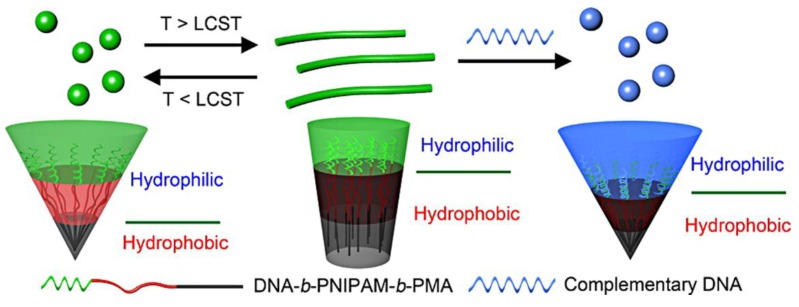
Self-assembly behavior of triblock copolymer of DNA-*b*-PNIPAM-*b*-PMA. Under LCST, the triblocks assembled into spherical micelles; above LCST, the spherical micelles transferred into cylinderic micelles. Further, the cylinder turned into spherical micelles by hybridizing with extended complementary DNA sequences. The green color indicate DNA strands, the red indicates polymer PNIPAM and the blue color stands for complementary DNA. Reproduced with permission from the authors of a previous paper [66]. Copyright 2016 American Chemical Society.

In 2016, Fujita and Meada explored the thermoresponsive structural transition of poly(*N*-isopropylacrylamide) (PNIPAAm)-*b*-DNA copolymers [65]. They studied the effects of salt and complementary DNA strand on the assembly process. When the temperature is above the lower critical solution temperature (LCST) of PNIPAAm, PNIPAAm turns insoluble in water. Above the LCST, the amphiphilic DNA copolymers assembled into dispersed spherical micelles at low concentration of salt whether the DNA segment was-single DNA strand or double strand DNA (dsDNA). If heating at high salt concentration, the hybridized copolymer firstly assembled into spherical micelles and further were aggregated due to the decrease of the repulsive interactions by the hybridization and addition of salt. The aggregated nanoparticles could be redispersed when the DNA duplex was denatured by heating. Subsequently, Park group reported the self-assembly behavior and multimodal morphology shifting of DNA-*b*-PNIPAM and DNA-*b*-PNIPAM-*b*-poly(methyl acrylate) (PMA) copolymers [66]. In Figure 5, the diblock copolymer DNA-*b*-PNIPAM possesses thermo sensitivity from PNIPAM and the molecular recognition properties of DNA. At above LCST, they assembled into spherical micelles. By hybridization and disassociation with complementary DNA-modified gold nanoparticles, it has achieved the aggregation and disaggregation of nanoparticles. DNA triblock copolymers of DNA-*b*-PNIPAM-*b*-PMA were composed of a hydrophobic block PMA, a thermo responsive block PNIPAM and an oligonucleotide. Interestingly, DNA-*b*-PNIPAM-*b*-PMA micelles were capable of transforming from sphere to cylinder and vise versa by taking advantage of temperature changes. The same morphology transformation existed while keeping the temperature above LCST through DNA hybridization.

### 3.2. Application of Amphiphilic DNA Block Copolymers

Amphiphilic DNA block copolymer assemblies possess potential application in drug delivery [62,67] due to its hydrophobic core of synthetic polymers for enveloping hydrophobic drugs and DNA corona for loading target molecules or nanoparticles. To overcome the problem of low cell internalization associated with the DNA block copolymer micelles, Mirkin group developed a strategy to prepare DNA-brush block copolymer micelles with a biodegradable core of polycaprolactone (PCL) and a dense layer of oligonucleotides as the corona [68]. Compared with the linear DNA block copolymer micelles, the DNA-brush block copolymer micelles exhibited a higher surface density of nucleic acids, a more negatively charged surface, a higher melting temperature, and a cooperative melting profile. It also showed more effective transfection agent-free cellular uptake and effective target gene expression in vitro. In 2017, Zhang Chuan and Zhang Ke further investigated the co-assembled micelles from DNA-*b*-PCL, PEG-*b*-PCL, and the PCL homopolymer (Mn = 10.5 kDa) [69]. The relationship between structures and properties was revealed in this system. These micelles also were able to realize high cellular uptake and effective antisense gene regulation.

**Figure 6:**
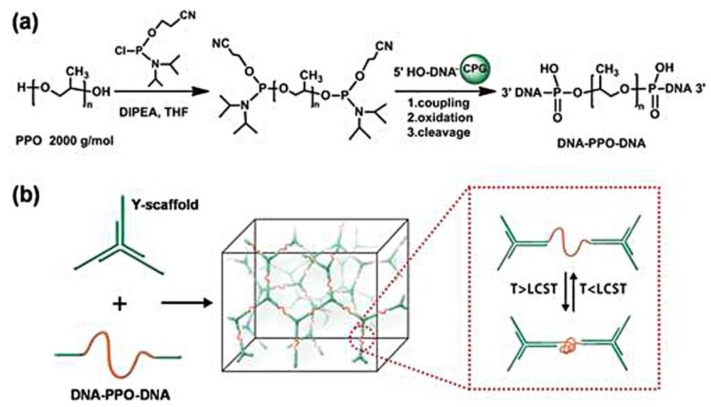
Illustration of the intramolecular collapse of PPO in a 3D DNA network. The solid phase synthesis of ssDNA-*b*-PPO-*b*-ssDNA (**a**) and the formation process of a 3D DNA network (**b**). The green color stands for DNA strands and the red color indicates PPO. Reproduced with permission from the authors of a previous paper [71]. Copyright 2017 John Wiley and Sons Publisher.

Besides the potential application in biomedicine, the amphiphilic DNA block copolymers were utilized as a component to construct more complex nanoarchitectures or higher ordered nanostructures. In 2014, Liu group took advantage of the frame-guided assembly strategy to fabricate size and shape controlled heterovesicles [70]. This system was composed of DNA modified gold nanoparticles, complementary DNA-*b*-PPO, and random DNA-*b*-PPO. DNA modified gold nanoparticles were regarded as frame. Complementary DNA-*b*-PPOs were the leading hydrophobic groups (LHGs), which were attached on the frame by DNA hybridization. After heating, the PPO segment became hydrophobic and further induced the random DNA-*b*-PPOsto assemble around the gold nanoparticle frame, and finally to obtain the heterovesicles. Their results suggested that different vesicles with controlled size and shape could be prepared by this approach and these vesicles could possess potential application in biomedicine. Furthermore, Liu group studied the intramolecular collapse of hydrophobic polymers of PPO in a 3D DNA network by using the triblock copolymer ssDNA-*b*-PPO-*b*-ssDNA, which were synthesized by solid phase method (Figure 6a) [71]. As shown in Figure 6b, ssDNA-*b*-PPO-*b*-ssDNA was the linker to connect the Y-scaffold by base pairing recognition. The results revealed that hydrophilic PPO was distributed uniformly in the 3D DNA network at low temperature, and hydrophobic PPO self-collapsed in the network at increased temperature. These results may provide reasonable explanation for nucleation-growing process of block copolymer.

Amphiphilic DNA block copolymers also can be coassembled with the diblock copolymers to form some new nanostructures. Park and colleagues demonstrated the use of a DNA block copolymer of polymethyl acrylate block-DNA (PMA-*b*-DNA) and a synthetic amphiphilic polymer of poly(butadiene)-block-poly(ethylene oxide) (PBD-*b*-PEO) to coassemble into giant polymersomes. The DNA strands were dispersed uniformly at the hydrophilic PEO shell. When these vesicles were connected through DNA hybridization, PMA-*b*-DNA amphiphiles migrated to the junction domain to form DNA islands within polymersomes. It also proved the effect of DNA islands on the DNA melting properties [72].

**Figure 7:**
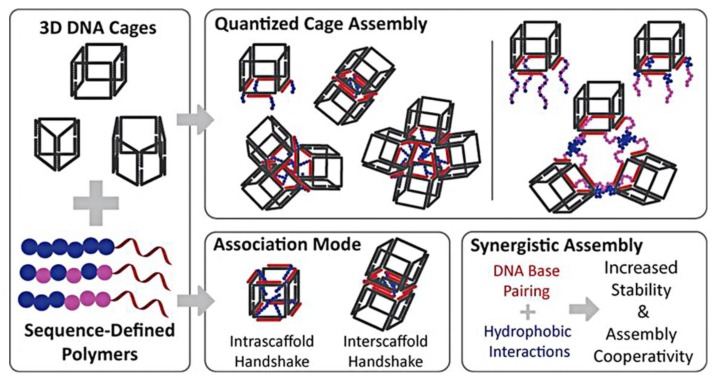
The self-assembly behavior of sequence-defined hydrophobic polymers on different shape of DNA cages. The black frame indicates DNA origami structures, the blue ball stand for long alkyl groups and the red line indicate roil DNA strand. Reproduced with permission from the authors of a previous paper [73]. Copyright 2016 American Chemical Society.

More interestingly, a new amphiphilic DNA polymer hybrid was introduced to combine with 3D DNA nanostructures and created highly complex nanostructures. In 2016, the Sleiman group reported the self-assembly of a sequence-defined hydrophobic polymers on DNA cages based on the synergistic action of hydrophobic interaction and precise base pairing interaction [73]. The sequence-defined DNA block copolymer was composed of long alkyl and oligoethylene glycol repeated units as well as oligonucleotide. As shown in Figure 7, DNA cages of different shapes were combined with these DNA block copolymers to generate a range of new nanostructures by tuning the number of long alkyl groups, the sequence order, and the orientation of the polymers. Hydrophobic polymers decorated on one face of the DNA cage resulted in the formation of monomeric cube, quantized cage assemblies, and “doughnut-shaped” DNA cage-ring structures by changing the polymer length and sequence order of polymers, respectively. Hydrophobic chains modified on both faces of the cage leaded to formation of DNA–micelle cages through an intrascaffold “handshake”. The DNA–micelle cages could encapsulate small molecules and possess better stability than the unsubstituted cage. These studies of new structural and functional DNA nanostructures provided an attractive approach to develop protein-inspired assembly modules in DNA nanotechnology. Subsequently, Sleiman developed a general strategy to transfer DNA patterns from a DNA cube to a polymeric nanoparticle assembled inside the cage in three dimensions. DNA-polymer amphiphiles were decorated at the sides of the DNA cage to form an internal hydrophobic domain. By covalently cross-linking, the DNA printed nanoparticles were generated inside the DNA cube. Due to the base pairing interaction, the DNA strands at the exterior of the polymer nanoparticles could hybridize with the complementary DNA modified nanoparticles to create highly complex nanostructures in a predictable manner. Furthermore, this method has potential application in targeted drug delivery and diagnostics due to the addressability and monodispersity of the resulting particles [74].

## 4. Amphiphilic DNA-Dendron Hybrids

Dendron with monodispersed molecular weight and precise nanostructure is regarded as an interesting building block to fabricate designed nanostructures. Obviously, DNA–dendron hybrids, as a new amphiphilic building block, can be utilized to prepare some ordered nanomaterials. But the first DNA–dendron assembly was not reported until 2010, because of its difficult in binding hydrophobic dendron and hydrophilic DNA molecules with the covalent bond. After the solving of the binding problem, the self-assembly and application in DNA nanoscience of DNA–dendron hybrids would attract great attention.

### 4.1. Self-Assembly of Amphiphilic DNA-Dendron Hybrids

**Figure 8:**
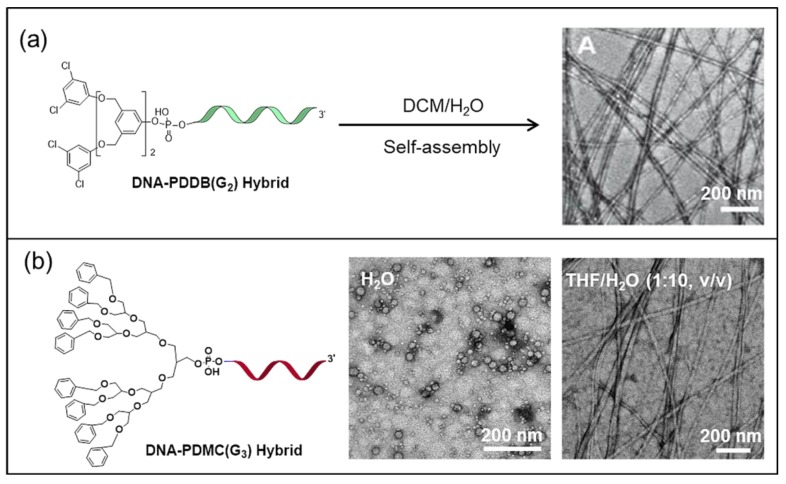
Self-assembly of amphiphilic DNA–Dendron hybrids. (**a**) Nanofibers from DNA and the second generation (G_2_) of dendron PDDB (poly(benzyl ether) dendron peripherally modified with dichlorobenzene) hybrids in dichloromethane and water mixture solution. The green segment indicates DNA strand. (**b**) Spherical micelles and nanofibers from DNA and the third generation (G_3_) of dendron–PDMC hybrids in water and THF/H_2_O (1:10, *v*/*v*), respectively. The red color of strand indicates DNA. Reproduced with permission from the authors of a previous papers [76,78].

In 2010, Sleiman and coworkers first reported the assembly behavior DNA–dendron hybrid consisted of hydrophilic OEG dendron and DNA strand in organic solvent [75]. Subsequently, Liu group investigated the self-assembly behavior of a series of DNA–dendron hybrids in aqueous solution [76,77,78]. There are two major kinds of dendritic molecular framework. One is hydrophobic poly(benzyl ether) dendron peripherally modified with dichlorobenzene (PDDB), the other is aliphatic polyether dendron (PDMC), as shown in Figure 8. DNA–PDDB hybrids with different generations of dendron and different lengths of DNA strand all self-assembled into nanofibers [76]. Further, the encapsulation of hydrophobic Nile Red molecules and the recognition of complementary DNA modified gold nanoparticles verified the assembling mechanism of the nanofibers that hydrophobic dendron is in the core and hydrophilic DNA is at the corona, respectively. About DNA–PDMC hybrids, in aqueous solution, they assembled into different nanostructures from spherical micelles to nanofibers, even to the irregular aggregates with the decrease of DNA length; when in a mixed THF/H_2_O (1:10, *v*/*v*) solvents, DNA–PDMC hybrids all assembled into nanofibers as changing DNA lengths or dendron generations [78]. The hydrophobic core of these nanostructures could encapsulate hydrophobic drug molecules and the corona DNA strands could load target molecules or nanoparticles by DNA hybridization, which suggested their potential application in biomedicine.

### 4.2. Application of Amphiphilic DNA–Dendron Hybrids

**Figure 9:**
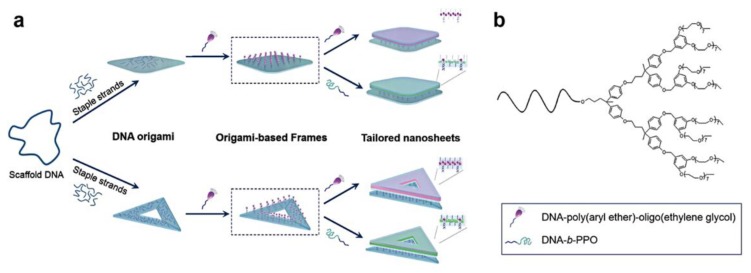
Illustration of the 2D nanosheets assembled from amphiphilic molecules on the DNA origami. (**a**) First, DDOEG molecules were anchored on the rectangular and triangular DNA origami structures by DNA hybridization, which leads to a high local concentration of hydrophobic molecules on the DNA origami surface. Subsequently, this surface works as a frame to guide the free hydrophobic molecules to assemble through hydrophobic effects, finally to generate continuous 2D nanosheets above the DNA origami. (**b**) The structure of DDOEG molecule. The purple molecule indicates DDOEG and the light green color indicates PPO segment. Reproduced with permission from the authors of a previous paper [82]. Copyright 2016 John Wiley and Sons Publisher.

Besides the self-assembly of the pristine amphiphilic DNA-dendron hybrids, the amphiphilic DNA–Denron hybrids can participate in multicomponent assembly. In 2014, Liu group demonstrated a frame-guided assembly method to fabricate heterovesicles with programmed geometry and dimensions [79]. They used DNA modified gold nanoparticle as the frame and the amphiphilic DNA–dendron hybrid as the leading hydrophobicgroups (LHGs). The DNA–dendron hybrid consisted of DNA strand and poly(aryl ether) dendron modified with eight oligo(ethylene glycol) (OEG) tails, named DDOEG. It attached at the frame by DNA base pairing interaction, and induced other amphiphilic molecules to assemble into heterovesicles with designed shape and size. It provided an effective strategy to prepare vesicles of controllable size and shape. In addition, amphiphilic DNA–dendron hybrids could be inserted into DNA Origami by DNA hybridization to induce amphiphiles assembled into higher ordered nanostructures. In 2015, Liu and coworkers achieved the self-folding of amphiphilic 2D DNA origami by using DNA–dendron hybrids DDOEG which not only hybridized with the DNA origami but also provided the hydrophobic driving force [80]. These results revealed that it was a simple and effective strategy to fabricate shape and size designable nanostructures with hydrophobic core and hydrophilic surface from DNA origami. Subsequently, the same group further undertook DNA origami scaffolds to displace the DNA modified gold nanoparticle as the frame. Through the same frame-guided assembly strategy, cuboid and dumbbell-shaped heterovesicles were fabricated [81]. This application demonstrated its potential to construct asymmetric and dynamic heterovesicle assemblies with complex DNA nanostructures. Almost simultaneously, Liu and colleagues also reported the use of single-layer rectangular DNA origami and triangular DNA origami as the frame, DNA–dendron hybrids DDOEG as LHGs to guild additional DDOEG or other amphiphilic DNA-*b*-polymers assembled into 2D nanosheet on the DNA Origami [82], as shown in Figure 9. It showed that the size and shape of the formed nanosheets was dependent on the arrangement of the LHGs on the DNA origami. Moreover, the nanosheets formed above the DNA origami could mimic the hydrophobic environment of biological membrane as well as provide the addressability for further implantation and study of the properties of membrane proteins.

However, DNA–dendron hybrid assemblies or DNA–dendron hybrid assembled nanostructures formed from 2D DNA origami have not been yet utilized in drug delivery up to now. The primary reason is probably that the assembled nanomaterials lack stability and the transfection rates are generally low. Recently, a DNA origami and dendron–protein hybrid complex system was introduced to enhance the stability and immunocompatibility [83]. The DNA origami was composed of 60-helix bundles. The dendron part of the dendron–protein conjugate possesses a positively charged domain that binds to the negatively charged DNA origami surface via electrostatic interactions. About the structure of the dendron–protein hybrid, two different proteins including bovine serum albumin (BSA) and class II hydrophobin (HFBI) and three different Janus dendrimers were investigated. The results revealed that the BSA, the second generation of Dendron (G2) coating, protected the DNA origami from the nuclease degradation, enhanced the transfection rate and significantly attenuated the immune reaction, which implied that this approach would extend the application of DNA-based nanostructures in drug delivery.

## 5. Conclusions and Prospective

The recent development of amphiphilic DNA organic hybrids (including small molecules modified DNA, DNA block copolymers, and DNA–dendron hybrids) to construct various nanostructures and be utilized as vehicles in biomedicine were reviewed in this paper (Appendix A, Table A1). Through hydrophobic interaction, amphiphilic DNA organic hybrids can self-assemble into different shape and functional DNA nanostructures, which have potential application in drug delivery and biomedicine. Moreover, these assembled DNA nanostructures possess hydrophilic DNA strands at the corona, which can load and carry inorganic nanoparticles or drug molecules due to its addressability. The challenge of the amphiphilic DNA nanostructures to be as smart drug-delivery vehicles in vivo is that how to enhance the stability and evade the immune response in higher organisms. Given that the strategies utilized in designing DNA nanodevices [84,85], the various protection and coating mechanisms such as virus protein [86], lipid membrane encapsulation [87], cationic polymer coating [88], or cationic dendron–protein coating [83] will be probably benefit for the circumvention of immune response. On the other hand, a variety of functional and smart DNA-based nanomaterials are fabricated by combining amphiphilic DNA hybrids with other 2D or 3D nanostructures, such as lipid vesicles and DNA origami. We believe that in the near future amphiphilic DNA hybrids can be introduced into more complex DNA scaffolds to develop bio-inspired systems and more smart nanosystems for biomedicine. More interestingly, it has the potential to be extended to dynamic DNA nanoarchitectures by using stimuli hydrophobic molecules. For example, stimuli amphiphilic DNA hybrids will serve as the switch of nanodevices such as nanopores [89,90], which is of particular importance for material transport and information transmission.

## Appendix A

**Table A1:** The self-assembly of the amphiphilic DNA–organic molecule hybrids and their application.

Category	Building Block	Assemblies	Application	Ref.
SMMD ^1^	Lipid-DNA Conjugates	Vesicles, micelles, liposomes	Cargo release, drug delivery	[23,24,25,26,27,28,29,30,31,32,53,54,55]
SMMD ^1^	Pyrene Modified DNA	Helical nanoribbons	Drug delivery	[35,36,37,38]
SMMD ^1^	PDI ^4^ Modified DNA	Supramolecular polymers, fibers	Construct novel structural motifs	[40,41,42,43,44,45,46,47]
SMMD ^1^	PpIX ^5^–DNA hybrids	2D nanocages	ROS generation	[48,49]
DBCs ^2^	DNA-*b*-PPO	Spherical micelles, nanofibers	Target drug delivery	[57,59,62,63]
DBCs ^2^	PPO-dsDNA-PPO	Large spherical micelles	Construct novel structural motifs	[64]
DBCs ^2^	DNA-*b*-PPO & AuNPs	Heterovesicles	Frame-guided assembly	[70]
DBCs ^2^	ssDNA-*b*-PPO-*b*-ssDNA and Y-scafford DNA structure	3D DNA network	Intramolecular collapse of PPO	[71]
DBCs ^2^	DNA-*b*-PNIPAAm;	Spherical micelles	Drug delivery	[65]
DBCs ^2^	DNA-*b*-PNIPAM-*b*-PMA	Spherical micelles and cylinders	Morphological control	[66]
DBCs ^2^	DNA-*b*-PCL, PEG-*b*-PCL and the PCL homopolymer	Spherical micelles	High cellular uptake and effective antisense gene regulation	[68,69]
DBCs ^2^	PMA-*b*-DNA and PBD-*b*-PEO	Giant polymersomes with DNA islands	Construct novel nanostructures	[72]
DBCs ^2^	Sequence-defined DNA block copolymer on 3D DNA cage	DNA cage-ring structures, DNA–micelle cages	Targeted drug delivery and diagnostics	[73,74]
DDHs ^3^	DNA–PDDB hybrid	Nanofibers	Drug delivery	[76,77]
DDHs ^3^	DNA–PDMC hybrid	Spherical micelles and nanofibers	Carry Nile Red molecules and positioning AuNPs	[78]
DDHs ^3^	DNA–DDOEG hybrid and gold nanoparticle	Heterovesicles	Biomimics and frame-guided assembly	[79]
DDHs ^3^	DNA–DDOEG hybrid and 2D or 3D DNA Origami	2D nanosheets, cuboid and dumbbell-shaped hetero-vesicles	Higher ordered nanostructures	[80,81,82]

^1^ Small Molecule Modified DNA; ^2^ DNA Block Copolymers; ^3^ DNA-Dendron Hybrids; ^4^ Perylenediimide; ^5^ Protoporphyrin.
